# Progress in Gynæcology

**Published:** 1887-07

**Authors:** C. B. Will

**Affiliations:** Chairman of the Committee on Gynæcology made to the Illinois State Medical Association


### PROGRESS IN GYNÆCOLOGY.

ABSTRACT OF THE REPORT OF C. B. WILL, M. D., CHAIRMAN OF THE COMMITTEE ON GYNÆCOLOGY, MADE TO THE ILLINOIS STATE MEDICAL ASSOCIATION.

THE progress in gynæcology during the past year has been essentially in the line of *reconsideration* and *revision*. Whilst, therefore, no event of very striking importance has occurred, judged from the sum total, as well as the practical character, of its presentations, it has perhaps been as prolific as any preceding. Old theories and methods have been overhauled and submitted to the test of comparative results, and new as well as old hedged about by the keenest enterprise and most searching criticism. The dazzling brilliancy of some former discoveries and practices has, as might have been expected, been dimmed somewhat in the light of further investigation, but the result has been, after all, to only give them a mellow, practical hue. Some recognized leaders in the profession have seemed to experience a radical change of view. Whilst Mr. Lawson Tait, for instance, announces with much self-confidence that he can diagnosticate a case of uterine disorder much better, or at least equally well, by the digital touch alone, or even by simply the history of the case, and that he almost never uses the sound or speculum, Dr. Emmet, not to be outdone, seemingly, goes him one better, to use the vernacular, and declares that for a long time he has not even *owned* a sound. At the last meeting of the American Medical Association a warm discussion was precipitated by the introduction of this subject in the section on gynæcology. A number of leading members stoutly condemned the use of the uterine sound under any circumstances, as entirely useless as a diagnostic agent, and actually criminal, in that it did much injury both mechanically and as a carrier of contagion, and the use of the speculum as well, for purposes of examination.

Such exhibitions are enough, certainly, to make the ordinary medical man stand aghast, and amidst the bewildering array of gynæcological paraphernalia with which, at the instigation of these same leaders, he has surrounded himself, wonder what it all means, and if progress has not at last turned backwards. In the minds of most medical men the question arises, Why this unseemly haste to appear in the front ranks of the retreating column? Is it because these instruments have been all the time useless? or that these men have from practice become so expert as to be able to do without them? If the former, then it is truly distressing, and augers evil for the future of so-called leadership, for, whilst it is an evidence of scientific candor and zeal to be ready to retreat from a position found to be untenable, or an opinion found to be erroneous, it is not a mark of well-balanced judgment to hastily discard methods which it has required generations to perfect. If the latter, then what can be more reprehensible than for one to try to pull from under the profession, so to speak, the ladder which has served to elevate one to a position of confidence and trust. In the midst of these throes of individual uncertainty, however, the mass of the profession have been going on in the even tenor of their way, realizing their ability and duty, as a whole, to put a check upon the exhuberance of their gifted but sometimes erratic *confreres*. They recognize the fact that revision does not mean annihilation, and that everything has a field of usefulness, however limited it may be.

Keeping in mind, then, the guiding stars of reconsideration and revision, it is curious to note the course which thought has principally taken in this branch of medicine and surgery. It is incumbent upon us to follow that course if we would know the points of a practical character developed during that period.

I shall, therefore, endeavor to group under three or four comprehensive headings, somewhat of the principal findings during the past year.

One of the few subjects which have given rise to much investigation and discussion is that of

PELVIC INFLAMMATIONS.

Generally speaking, by pelvic inflammation is meant pelvic peritonitis and cellulitis. At least that is in accordance with the evidence deduced from the current literature of the subject. The question of the etiology of this affection, or these affections, is yet *sub judice.* Many hold that inflammation of the pelvic cellular tissue depends primarily almost entirely upon disease of the ovaries or tubes; that, as Dr. Battey says, "a pelvic cellulitis which gives rise to so much trouble is, in a large proportion of cases, secondary and not primary." This would seem to be largely, at least, the case, for aside from the generally recognized influence of pressure and other injuries in child-birth, much evidence has been advanced to prove gonorrhœa a very potent cause. The constantly accumulating evidence of this latter as an active agent in the production of destructive disease of the tubes, as well as the known laws of septic communicability, would seem to favor this presumption. By far the most fruitful primary source of these affections, however, in the estimation of the writer, is the development of bacteria within the vagina, as a result of the introduction in one way or another of pathogenic germs. There is, as has been stated by a recent writer, a great deal of criminal carelessness in infecting patients by dirty finger-nails and instruments. It is indeed surprising to one who has not given the subject special attention, with what ease and readiness a patient may be seriously infected in this way, and especially during or immediately subsequent to operations in the genital tract. Dr. E. W. Cushing, in a recent communication, very truly says on this subject: "The same principle (that of antisepsis) is to be further applied to pelvic imflammations of other than puerperal origin, and reasoning from certain premises, which may be now accepted as axioms, a system of treatment and procedure is in evolution for the avoiding of those so-called accidents, by which, after the slightest gynæcological operation, 'takes cold,' and 'cellulitis' develops. Now, 'catching cold,' with a subsequent inflammation of the pelvis, means septic infection.

Cellulitis means either a direct infection of the lymphatics, from the surface laid bare by operation, or, far more frequently, an infection of the endrometrium, which passes up the tubes, and sets up a peritonitis. This is happily unusually localized, and may not give rise to suppuration, but sometimes unhappily the process cannot be controlled, the patient dies, and it begins to be pretty hard to persuade the friends that she "caught cold" because the nurse left the window open, or lifted the blanket incautiously.

The immediate cause, or, if one will avoid that word, the constant antecedent and inseperable concomitant, of all such inflammations is an infection with living bacteria or germs, which, differing in their nature, and falling on soil more or less favorable, give rise to the greater or less severity of symptoms; from a light shiver, moderate fever, and local hardness, all the way to an explosion of peritonitis or septicæmia with speedy death.

At present we can do very little to check, or control, such processes when they have passed into the lymphatics or the fallopian tubes. The graver cases are called septic, the lighter inflammatory; but the nature of the process is the same as in any case—a bacterial infection.

We do not always know at first when cellulitis is present, and "in every stage," says Dr. Jackson, "we feel doubt as to whether this disease or some other is present. Sometimes, even when it gets on to its later stages, we have doubt." Emmet, in his paper before the American Gynæcological Society, says: "Inflammation of the connective tissues between the rectum and vagina behind, and in front of the uterus, bladder and ureters, is generally accompanied by phlebitis, and is a frequent consequence of the pressure made during childbirth, but it is a matter of common observation that this form of pelvic inflammation tends rapidly to resolution, and the tissues soon regain their healthy state if septic poisoning does not take place. With the introduction of septic matter from any source, the lymphatics become inflamed and the peritoneum is rapidly involved." A report from the clinic of Sanger, of Leipsic, by Dr. H. Bigelow, of Boston, says as to a differential diagnosis that peritonitis is frequently found unassociated with any inflammation of the connective tissues, but cellulitis generally involves the peritoneum, and that in general those exudates which lie behind the uterus are of the peritoneum, whilst those on the sides, which take origin in the connective tissues of the liamentum latum and which may stretch from the fassa iliaca to the kidney, are cellulitic. Dr. James B. Goff, in a paper before the Alumni Association of the Women's Hospital, N. Y., voicing the sentiments of a large number, says that "in the light of the knowledge yielded us in late years, and the findings during laparotomy, he has come to the conclusion that pelvic cellulitis has been dethroned from the prominent position which it has held in uterine pathology, and that in its place must be substituted salpingitis and peri-salpingitis, oöphositis and peri-oöphositis, peri-uterine adenitis, peritonitic bands and adhesions. When cellulitis as such occurs, it is acute in its nature and harmless in its action, for it leaves no trace behind such as may be detected by the pathologist. The small indurations around the uterus, deemed by Emmet and others to be evidence of cellulitis, have been unquestionably shown to be the result of peritonitis. Large exudations around or to one side of the uterus are due to plastic peritonitis. These exudates may of course either be absorbed or break down into pus, in which latter event we have pelvic abscess."

As to the treatment of these affections during the acute stage nothing new seems to have been offered, the, to some, doubtful remedial influence of opium, quinine, hot douches and poultices being allowed the field.

The treatment of the results of acute pelvic inflammations has, however, met with the wide-spread and careful consideration which it unquestionably deserves, although to the true scientist and conservative practitioner the necessity for it seems a melancholy commentary on the boasted therapeutics of the day. Abscess is one of the most frequent conditions or results calling for interference, and it is at the same time the most urgent. The means of diagnosis and treatment are measurably intertwined. The instrument of the former is oft times the agent of the latter. The aspirator is now generally considered a useful aid in diagnosis, as well as curative agent. Says a recent writer: "When a large mass bulges down behind the uterus and cannot be felt in either hypogastrium, we may affirm that it is not an abscess in the cellular tissue. It may be pus within the peritoneum, but usually it is the distended fallopian tubes which are felt. Here aspiration can only harm," and this is considered almost the only exception. "Laparotomy and removal of the tubes are then, of course, requisite." Evacuation of the pus is, of course, the only curative measure in pelvic abscess; the question being only how best to accomplish it in a given case. Dr. W. H. Byford thinks it should be done by laparotomy only when supra pelvic or intra-peritoneal. Dr. Christian Fenger, of Chicago, in a discussion of this subject before the Gynæcological Society, referred to the condition when a communication exists between an abscess and the intestinal tract, and remarked that evacuation of the pus into the bowel is sometimes followed by spontaneous recovery; but, as a rule, such condition is extremely dangerous, for the abscess cavity is constantly being infected with septic material from the intestine. He quotes as authorities Pean, Schrœder and Emmet, and concludes that under such circumstances the patient is in great and constant danger of dying, and therefore an operation should be done, if possible, to provide for drainage of the abscess, and that can best be done by laparotomy. In commenting on Dr. Byford's criticism and suggestion that drainage under such circumstances should be secured through dilatation of the rectum, or rather sphincter ani, Dr. F. said that he had never heard of the idea before, but that as such openings were often narrow and tortuous, and the neighboring organs immovable, drainage could not then be satisfactorily effected, and to cut into the fistulous tract in that locality would be dangerous in the extreme.

In a letter to Dr. Jaggard, of Chicago, Dr. Paul Mund writes of this question: "I do not agree with Prof. Byford's rectal treatment, as a rule, and certainly have not in my quite extensive experience found true laparotomy required to evacuate and drain a pelvic abscess. I mean opening the peritoneal cavity. Neither has my experience been that of Dr. Dudley, who says that the mortality, from abscess opening into the rectum, is great."

Dr. H. T. Byford had, however, effected a cure in that way, and your reporter can add his testimony to its efficiency in at least one case, a few months since, which it is not necessary to report in detail, but which presented an opening about two and a half inches above the anus, and through which a catheter was introduced and drainage effected.

After the dilatation of the sphincter ani no special difficulty was experienced either in finding the opening or introducing the instrument. Tolerably strong injections of carbolic acid solution were used daily, and although some considerable irritation of the rectum was produced by the manipulation, the abscess cavity gradually closed up and the frequent irregular periodic discharge entirely ceased, and I believe the patient to be well.

As one of the best expositions of the operative procedures looked upon with favor at present, and on account of its succinctness, I append the following *resume* and review of a paper by Dr. Imlach (*Pacific Medical and Surgical Journal: Journal of Obstetrics*):

"The writer takes the ground that an abscess in the pelvis, more than in any other locality of the body, should be opened, emptied and drained, because in the pelvis a collection of pus is not likely to remain localized, but may burrow freely, burst into the cellular tissue, perforate the peritoneal cavity with fatal result, or empty into the bladder or rectum. These possible results it should be aim of good surgery to prevent by free incision and drainage. It should always be remembered, however, that to cure a pelvic abscess, drainage is not always necessary. Aspiration will frequently suffice. (Two instructive cases are recorded where the abscess was exposed by laparotomy and then aspirated, and cure was immediate and permanent.) When drainage of the abscess is aimed at, the peritoneal cavity ought not to be opened. In suitable cases, Imlach considers it safe to open up the inguinal canal, pass a probe along the swollen round ligament into the abscess, insert a glass or rubber drain-tube, and thus a large cavity may be drained without fear of peritonitis. Where an abscess seems to point in the inguinal region, its tendency is not to burst, but to extend toward the anterior iliac spine. Constant poulticing will frequently not make such an abscess open, but the incision just described will efficiently drain and cure the case. (Cases where this method was resorted to are noted, and in one the result was satisfactory, although unsuccessful aspiration *per vaginam* had been tried.) In case of an abscess occupying a large area of subperitoneal tissue, and reaching to the ribs, an incision above the anterior superior spine will shorten the suppurative process. Such an incision may be extended inwards along Ponpart's ligament, or outwards above the iliac crest. By such an incision likewise perityphlitic and psoas abscesses are drained. Through only a small incision a large area may be drained. Reference is then made to aspiration *per vaginam*, as also *per rectum*, an instance of the latter being recorded as ineffectual, and followed by laparotomy and eventual recovery. As for the indications for aspiration, Imlach makes no positive assertion. If the aspiration fails, it is better to make a free opening at once. He has but a limited confidence in the aspirator, but yet thinks that where harm has resulted from its use it is usually because the needle has been introduced into an ovarian or tubal abscess—'a procedure of infinite risk and never ultimately successful.' 'A free vaginal incision over the most prominent part of the tumors with (or without) insertion of the finger and breaking up of adhesions has been recommended.' 'I must confess I prefer almost any other method. The tumors which, by their vaginal prominence, without adominal swelling, suggest this treatment, are either pelvic hematocules or other tubal disease, and to make a free opening into them through the vagina is to court fatal peritonitis.'" The writer (E. H. G.) then adds: "Herein Imlach, we believe, is in error. A pelvic abscess may bulge posteriorly to the uterus and not be felt through the abdomen, and still be safely opened, the cavity curetted, if need be, and the opening enlarged by the finger. See, in this connection, Mundè, on Pelvic Abscess. *American Journal of Obstetrics*, 1886. We further believe that free opening, evacuation and drainagé of an hematocele *per vaginam* is by no means bound to result in fatal peritonitis. If the blood effusion be encysted, whether it was originally intra or extra-peritoneal, matters little; provided it present distinctly in the vagina, it may safely be opened, cleaned, and, if need be, drained. As for the expectant treatment of pelvic abscess, Imlach does not favor it, for in the face of such treatment the patient's health is apt to become permanently injured.

"The expectant treatment lives not on its own successes, but on the occasional failure of surgery in tuberculous subjects. By early opening of the abscess, caseation of the lumbar lymphatic glands may probably be prevented. When opening is long delayed speedy death is inevitable. Another comprehensive subject which has kept the profession on the qui vive for the twelve months just passed, as well as the preceding, is that of

REMOVAL OF THE UTERINE APPENDAGES.

About this subject, more, perhaps, than any other, there prevails a sentiment of mistrust and apprehension. That the so-called "Tait's operation," or its modifications, has been abused, no one will care to dispute, and the tide of criticism has turned strongly in upon it. That the operation has frequently been done unnecessarily, even by those who can show brilliant records as operators, is held by the mass of thinkers in the profession. The excuse of many others who have essayed the operation can be considered little short of the novelty of the proceeding. There seems to be a sort of weird fascination about it which attracts men beyond the dictates of their better judgment. That there is a field for the operation would seem now to be beyond question, but that it is far more closely limited than the practice would seem to indicate is equally true. Dr. T. A. Emmet, in a recent paper, says: "The average success in abdominal section is not equal to the average danger to life," and concludes as follows: "I believe that the operation is practiced too often, even by those who have the smallest death-rate, and I predict that five years will not pass before it will be necessary to offer an apology when its performance is suggested. The operation doubtless fills an important place in gynæcological surgery, but its usefulness must be more closely defined, and its practice greatly restricted, or the good name of the profession will surely suffer in the future." Dr. Joseph Price, of Philadelphia, claims that Mr. Tait does not guess at conditions, and resort to abdominal section for diagnostic purposes as recklessly as is generally supposed, and through which statements a world of mischief has already come. Tait says himself, on this subject: "Save when the seat of such organic disease as will explain genuine suffering, the uterine appendages ought not to be removed, and that those who attribute all the pelvic aches and ails of women to the ovaries and tubes, and rush to remove them, are dangerous people."

It is of the utmost importance that all cases which come under our notice should receive a more rigid examination, to the end that diagnostic signs of a more positive character be discovered, and make our interference more a matter of scientific certainty. A correct diagnosis is to-day the prominent factor in solving the problem of abdominal section in any given case. Now and then, during the discussions of this subject during the year, a point or two of diagnostic value has been brought out. In a discussion of this subject before the Obstetrical Society of Philadelphia, the fact was pointed out that a very important symptom in disease of the tubes is frequently a stillicidium of bloody grumous material per uterus and vaginum, which is regarded by the patient as a prolonged menstrual period. This undoubtedly flows from the tube, and is altogether analagous to the free purulent discharge from the tubes of pyosalpinx, and it might be wise to catheterize the tube and dilate and endeavor to relieve a case of hemato or pyo-salpinx in this way if the discharge exists in sufficient quantity to lead to a suspicion of a patulous uterine orifice.

A further aid to diagnosis may be found in the history of the case as to the presence at any time of a gonorrhœa, as it is pretty well established that gonorrhœa is a very frequent source of chronic and destructive disease of the tube and ovary, especially pyo-salpinx.

Dr. Howard Kelly places his reliance regarding diagnosis entirely in a skilled bimanual examination, by which he always accurately maps out all the peculiarities of the case before operation. If there is rigidity and resistance, it is necessary to etherize, but he has yet to see the case, when the presumptive signs were those of tubal and lesser ovarian disease, where the structures could not be picked up between the two hands and outlined. Introducing the finger as high as possible, by forcing the hand well under the pubic arch, and carrying the sensitive pulp up against the post fornix or either lateral fornix, and then playing up and down with the other hand pressing on the abdomen, and creeping a quarter inch at a time without ever fully relaxing, and letting structures in between roll through the two fingers, and in case of an ovary, running round its whole periphery, or of a tube, tracing it up to the cornu uteri and down into the retro-uterine pouch, where it usually terminates, gives often most surprising results, and would doubtless, if universally carried out, change hundreds of diagnoses of leucorrhœa, endometritis, and flexions with adhesions, to the far more serious ones of pyo or hemato-salpinx."

Dr. W. G. Wylie, of New York, in a recent report, says on this subject: "Repeated attacks of local peritonitis, or a constant burning pain over the appendage, which is at times rendered rather worse than better by any form of local treatment, is perhaps the most reliable indication that removal is the only way of giving relief."

As regards the *tecnique* of the operation for removal of the uterine appendages, nothing has been offered that physicians are not familiar with in connection with the general subject of laparotomy, except, perhaps, the use of hot water within the peritoneal cavity to prevent shock. In the report above referred to, Dr. Wylie, in the accuracy of whose observations and judgment the writer has great confidence, gives an account of his use of hot water in this connection, and his experience on another point or two, in the following statements:

*First*—That hot water cannot only be safely used to wash out the peritoneal cavity, and that it is a simple and efficient hemostatic for oozing the points too numerous and small to tie; but that in prolonged operations, and immediately after the operation, free irrigation of the peritoneal cavity with water at 105° to 110° is a powerful and efficient agent, it lessening, if not preventing, the effects of shock. Towels wrung out in hot water have been used to protect the intestines when turned out of the cavity; but I do not know that hot water irrigation of the peritoneum for shock has ever before been suggested. The temperature of the hot water must not exceed 110° or it may cause shock.

*Second*—That the tympanitis and vomiting and other symptoms supposed to be due to septic peritonitis after laparotomy are best overcome by enemas, and if these fail, by a quick purgative. That in these cases, at least, the bowels should be moved, not kept constipated, as has been generally practiced. That it is probable that in many cases the persistent and exhausting vomiting may be the direct result of intestinal obstruction, the result of bands of adhesions constricting the intestines. For a long time I have seriously doubted the existence of septic peritonitis in some cases where the vomiting preceded the rise of temperature and other symptoms of sepsis. By experience I found it best to move the bowels whenever indicated by tympanitis or vomiting, even during the first twenty-four hours after the operation—of course making a distinction between these symptoms and those due to the effects of ether. The vomiting from ether is likely to be preceded by a marked nausea, and is usually violent and gagging in character, while that from obstruction is passive and more like eructation. The quantity vomited is large, and at first brownish colored, and attended with extreme exhaustion and marked tympanitis. Without doubt the opium treatment taught by Dr. Alonzo Clark has proved to be the best plan for limiting local peritonitis, and when carefully carried out may prevent death in some cases of general peritonitis; but I am certain that it is not best suited for septic peritonitis following laparotomy. I usually give enough opium or morphine to relieve pain and restlessness, and keep the respiration down to 16, or even 12 in some cases. But when tympanitis or eructive vomiting begins, I at once move the bowels by turpentine or oxgall enemas, or by a seidlitz powder or Rochelle salts.

I took the hint from a remark of Mr. Lawson Tait that a brisk purgative would cure septic peritonitis, but the method of effecting it and the explanation as to preventing intestinal obstruction are my own.

Much interest has been manifested during the past year on the subject of tamponade of the vagina for the treatment of various diseased conditions of the uterus and adnexa. The "new departure" in this line of Dr. Engleman, of St. Louis, described by Dr. J. H. Etheridge at the last meeting of this society, has been since used with gratifying success by the latter, who has, however, substituted "antiseptic wool" for the cotton used by the former, and finds it to be of much greater utility for every purpose for which the tamponade is practiced. The dry dressings thus applied, your reporter, too, has found to be of great value in the treatment of not only vaginitis of obstinate character, but those distressing cases of vesical irritation arising therefrom, as well as from depression of the weighty uterus and appendages, and in restoring the pelvic circulation in cases of general engorgement, thereby relieving and promoting the cure of congestive and chronic inflammatory trouble and misplacements. On this subject Dr. Thornton Parker, of Newport, R. I., has the following to say: "For prolapsed and sensitive ovaries, for the treatment of prolapsed uterus, for many forms of misplacement, and in cases of leucorrhœ, marine lint is much preferable to absorbent cotton, and keeps sweeter and cleaner longer than any other appliance. The support it affords and the sense of comfort it induces make it very acceptable to patients. I apply it always in a ring form, introducing it through the speculum. Where a large pessary is indicated, as in prolapsed uterus, it must be introduced without the speculum. The oakum never irritates, and is always free from the odor following the use of any other pessary or of absorbent cotton. The oakum pessary not only absorbs and disinfects the vaginal secretions, but does not lose its place, and continues to afford support until removed. This is not the case with absorbent cotton, which is very objectionable in most instances. In cases of anteversion or retroversion as well as in cases of prolapse, the oakum pessary will be found superior to the hard or soft rubber pessary. The oakum is not only an excellent support, but possesses healing properties when applied to the diseased mucous membrane of the vagina. I usually saturate the oakum pessary with *boracine.* This improves the wholesome action of the pessary and permits its being retained for a much longer time.

The following periodicals were consulted in making out this report: *American Journal of Obstetrics; Journal of the American Medical Association; New York Medical Journal; Medical Record; Lancet and Clinic; Medical News; New England Medical Monthly; Medical Analectic; London Lancet; St. Louis Medical and Surgical Journal.*

